# Hepatic iNKT cells produce type 2 cytokines and restrain antiviral T cells during acute hepacivirus infection

**DOI:** 10.3389/fimmu.2022.953151

**Published:** 2022-09-09

**Authors:** Svjetlana Raus, Jarrett Lopez-Scarim, Joshua Luthy, Eva Billerbeck

**Affiliations:** ^1^ Department of Medicine, Division of Hepatology, and Department of Microbiology and Immunology, Albert Einstein College of Medicine, Bronx, NY, United States; ^2^ BD Life Sciences - FlowJo, Ashland, OR, United States

**Keywords:** T cells, NKT (natural killer T) cells, type 2 immune response, mouse model, hepatic immune regulation, viral hepatitis, hepatitis C virus infection

## Abstract

Chronic hepatitis C virus (HCV) infection is a curable disease, but the absence of a vaccine remains a major problem in infection prevention. The lack of small animal models and limited access to human liver tissue impede the study of hepatic antiviral immunity and the development of new vaccine strategies. We recently developed an immune-competent mouse model using an HCV-related rodent hepacivirus which shares immunological features with human viral hepatitis. In this study, we used this new model to investigate the role of invariant natural killer T (iNKT) cells during hepacivirus infection *in vivo.* These cells are enriched in the liver, however their role in viral hepatitis is not well defined. Using high-dimensional flow cytometry and NKT cell deficient mice we analyzed a potential role of iNKT cells in mediating viral clearance, liver pathology or immune-regulation during hepacivirus infection. In addition, we identified new immune-dominant MHC class I restricted viral epitopes and analyzed the impact of iNKT cells on virus-specific CD8+ T cells. We found that rodent hepacivirus infection induced the activation of iNKT cell subsets with a mixed NKT1/NKT2 signature and significant production of type 2 cytokines (IL-4 and IL-13) during acute infection. While iNKT cells were dispensable for viral clearance, the lack of these cells caused higher levels of liver injury during infection. In addition, the absence of iNKT cells resulted in increased effector functions of hepatic antiviral T cells. In conclusion, our study reports a regulatory role of hepatic iNKT cells during hepacivirus infection *in vivo*. Specifically, our data suggest that iNKT cells skewed towards type 2 immunity limit liver injury during acute infection by mechanisms that include the regulation of effector functions of virus-specific T cells.

## Introduction

Inflammatory liver diseases such as chronic viral hepatitis are a leading cause for the development of liver fibrosis and hepatocellular carcinoma (HCC) and consequently represent a major global health problem (1, 2). 58 million people worldwide are chronically infected with hepatitis C virus (HCV) (3). Recently developed directly acting antivirals (DAA) against HCV now cure the majority of treated patients (4). However, a prophylactic vaccine which is likely needed for global HCV eradication still does not exist (5). The hepatic immune system is thought to play both, protective and detrimental roles in inflammatory liver diseases (6, 7). While hepatic immune responses are essential for the clearance of HCV infection, they also contribute to liver injury and to the progression of liver disease in viral hepatitis (8, 9). A better understanding of the mechanisms that regulate the development of an effective antiviral immune response and the balance between tissue damage and protection during inflammation is a prerequisite for the development of HCV vaccination strategies and therapeutic options for the treatment of advanced liver disease.

As a major metabolic organ of the body the liver constantly receives gut-derived blood rich in nutrients, toxins and bacterial products through the portal vein (7). This direct exposure to harmless microbial and dietary antigens creates a unique tolerogenic immunological microenvironment enriched in various innate immune cells, innate-like lymphocytes and T cell subsets (6). It is well established that CD4+ and CD8+ T cells are crucial for the clearance of HCV (10). However, the role of distinct hepatic T cell subsets or innate-like lymphocytes, such as natural killer T (NKT) cells, and their respective effector functions (e.g. type 1 or type 2 cytokines) in mediating viral clearance, immunopathology, immune-regulation or tissue repair is little defined.

NKT cells are a tissue-resident lymphocyte subset enriched in the liver (11–13). They recognize foreign- or self-lipid antigens presented by the MHC class I-like molecule CD1d (11, 14). Hepatic NKT cells consist mostly of invariant NKT cells (iNKT cells) which express a semi-invariant TCR and react to glycosphingolipids (e.g. αGalCer) (15, 16). However, iNKT cells can also be activated directly by cytokines, e.g. IL-12, IL-18 or IL-25, in a TCR independent manner. iNKT cells are functionally highly heterogenous with a subset diversification (based on transcription factor and cytokine expression patterns) similar to CD4+ T helper cell subsets: NKT1 (Tbet+PLZF-, IFN-γ), NKT2 (GATA-3+PLZF+, IL-4, IL-13), NKT17 (RoRγt+, IL-17) or NKT10 (IL-10) (13–16). Upon activation, iNKT cells rapidly secrete pro- or anti-inflammatory cytokines depending on their polarization in a specific tissue environment (13, 17). This property gives them significant immune-regulatory capacities.

Hepatotropic viruses do not express lipid antigens, however they can induce the production of IL-12 or IL-18 and can significantly alter hepatic lipid metabolism, which may lead to modified self-lipid presentation in the liver (15, 18). In fact, some studies suggest activation, proinflammatory and antiviral functions of NKT cells in HCV and hepatitis B virus (HBV) infection (18–21). Interestingly, in a mouse model of sterile liver injury self-lipid recognition activated iNKT cells to become IL-4 secreting NKT2 cells which mediated resolution of inflammation and tissue repair (22). These and other recent studies (23–25) highlight the diverse roles NKT cells can play in the inflamed liver. The exact role of these cells in the liver during viral hepatitis *in vivo* however is not known.

A major limitation in the study of hepatic antiviral immune mechanisms is the lack of suitable animal models and limited access to human liver tissue (26). Most hepatitis viruses, including HCV, have a narrow host tropism to the human liver. Fully immune-competent small animal models for these viral infections do not exist (26). However, we have recently developed an immune-competent mouse model based on an HCV-related rodent hepacivirus isolated from Norway rats of New York City: Norway rat hepacivirus (NrHV) (27, 28). NrHV infection in mice shares important virological and immunological features with HCV infection in humans such as exclusive viral hepatotropism, T cell dependent clearance, immune-mediated liver injury and susceptibility to reinfection (28). This novel model now allows for the mechanistic study of antiviral hepatic immunity during a hepacivirus infection *in vivo*.

In this study, we used the NrHV mouse model to investigate the role of hepatic iNKT cell subsets during acute infection. Our data revealed the induction of a heterogenous iNKT cell response during infection which, surprisingly was dominated by the activation of iNKT cells with a mixed type 1/type 2 immune signature and pronounced production of type 2 cytokines, such as IL-13 and IL-4. The absence of iNKT cells during infection resulted in increased acute liver injury and elevated effector functions by virus-specific CD8+ T cells. In summary, our study suggests a new type 2 immunity associated regulatory function of hepatic iNKT cells during hepacivirus infection *in vivo.*

## Material and methods

### Mice

C57BL/6 and B6.CD1d^-/-^ mice were purchased from the Jackson Laboratory. All mice were bred and maintained under specific pathogen-free conditions at the Institute for Animal Studies of Albert Einstein College of Medicine. Mice were kept on a 12h light/dark cycle with food and water ad libidum. All experiments with mice were in accordance with the NIH Guide for the Care and Use of Laboratory Animals and approved by the Albert Einstein College of Medicine Institutional Animal Care and Use Committee. 8-week-old female and male mice (at equal numbers) were used for experiments. For reinfection experiments age-matched control mice were used. All experiments in this study were performed, at least, in duplicates at two independent time points using 4-8 mice per group.

### Virus

Norway rat hepacivirus (NrHV), an HCV-related rodent hepacivirus, was used for mouse infection experiments (27, 28). The viral inoculum used in this study was a cDNA clone-derived virus stock based on the mouse adapted NrHV clone B sequence that has previously been described (28).

### Infection of mice

Mice were infected intravenously with 10^4^ NrHV genome equivalents (GE) by retro-orbital injection. For secondary challenge experiments mice were infected using the same route of infection and the same inoculum as for the primary infection (28).

### RNA isolation and NrHV RT-qPCR

Viral RNA from mouse serum was isolated using the High Pure Viral Nucleic Acid Kit (Roche). Mouse serum and RNA were stored at -80°C. NrHV RNA was detected and quantified by one-step RT-qPCR using TaqMan Fast Virus 1-Step Master mix (Applied Biosystems) at a Quantstudio 12kflex (Applied Biosystems) with the following protocol: 50°C for 30 min, 95°C for 5 min followed by 40 cycles of 95°C for 15 sec, 56°C for 30 sec and 60°C for 45 sec. The sequences of NrHV E1 specific primers used for this protocol were:

Sense: GGCTGTGTCATCTGCGAGCA,

Anti-sense: CGACGAAGTCTATATGGTGGGC

Probe: [6-FAM]GGCCCCATGGTATCCAGGTCACCGCACTA[BHQ1a-6FAM]

An NrHV standard curve was generated from *in vitro* transcribed RNA from a plasmid encoding the partial NrHV E1 sequence. After *in vitro* transcription, the RNA was quantified and diluted to concentrations ranging from 10^8^-10^1^ GE/µl.

### Leukocyte isolation

Mice were sacrificed at multiple time points post infection (pi) and leukocytes were immediately isolated from blood, liver and spleen. Blood-derived leukocytes were isolated from heparinized blood by Ficoll-density gradient (Cellgro) centrifugation (20min, 2000rpm, 20°C). The liver was perfused with cold PBS (Gibco) prior to leukocyte isolation. The perfused liver tissue was minced and digested with collagenase IV (HBSS, 0.01% collagenase IV, 40mM HEPES, 2mM CaCl_2_ and 2U/ml DNAse I) for 20min at 37°C followed by homogenization through a 100µm cell strainer (BD Biosciences). Leukocytes were subsequently isolated from liver cell suspension by Ficoll-density gradient centrifugation (20min, 2000rpm, 20°C). The spleen was minced and digested with collagenase IV (HBSS, 0.01% collagenase IV, 40mM HEPES, 2mM CaCl_2_ and 2U/ml DNAse I) for 10min at 37°C. A leukocyte suspension was prepared from the digested spleen by homogenization through a 100µm cell strainer. Isolated leukocytes from blood, liver and spleen were washed twice in PBS and directly analyzed.

### Flow cytometry

For surface marker staining, cells were plated in PBS on a 96-well-plate, blocked for 10 min at RT with anti-mouse Fc-block (BD Biosciences), stained with appropriate antibodies for 15 min at RT, washed twice with staining buffer (PBS with 1% FCS) and fixed with 4% paraformaldehyde. For tetramer staining cells were incubated with the appropriate tetramer in staining buffer for 15 min at 37°C prior to surface staining. For staining of intracellular proteins, cells were first stained for surface markers followed by permeabilization (15min, 4°C, Transcription Factor Staining Buffer Set, eBioscience) and staining for intracellular proteins (30 min, 4°C). For staining of intracellular cytokines produced by iNKT cells, cells were stained immediately after isolation as described above for surface marker and intracellular proteins. For staining of intracellular cytokines produced by T cells, cells were plated in RPMI (Gibco) and stimulated with 5ng/ml PMA and 500ng/ml Ionomycin (Sigma) in the presence of brefeldin A (eBioscience). After 5h of incubation at 37°C cells were stained for surface markers, permeabilized (Transcription Factor Staining Buffer Set, eBioscience) for 15 min at 4°C and stained for intracellular cytokines (30 min, 4°C). For the analysis of total cell numbers CountBright absolute counting beads (Molecular Probes) were used according to the manufacture’s instruction. FACS analysis was performed using a Cytek Aurora analyzer (Cytek). Spectroflow (Cytek) and Flow Jo Version 10 (BD Biosciences) software were used for data analysis.

### Identification of MHC class I NrHV epitopes and virus-specific CD8+ T cell stimulation

In order to identify MHC class I restricted NrHV epitopes we obtained 18mer (11aa overlap) peptides (Mimotopes) covering the entire NrHV clone B sequence (28). Using this overlapping peptide library we performed IFN-γ Elispot assays with CD8+ T cells from C57BL/6 mice at day 14 and 21 post NrHV infection. Positive responses towards 18mer peptides were confirmed by intracellular cytokine staining (ICS) as described above. Specifically, we isolated leukocytes from blood and liver of mice at day 14 and 21 pi and stimulated the cells with 5 μg/ml of the respective 18mer peptide in the presences of brefeldin A for 5h at 37°C followed by intracellular staining for IFN-γ and TNF-α. We then performed IEDB (Immune Epitope Database) analysis to identify predicted MHC class I restricted minimal epitopes within the 18mer peptides. We tested multiple shorter peptides for each predicted epitope using ICS. With this method we identified two immune-dominant epitopes in the nonstructural proteins NS3 and NS4: H2Kb restricted NS3_1043-1052_ CTEFYLATRL and H2Db restricted NS4_1602-1611_ SAALNPAPEM. MHC class I tetramers loaded with these epitopes were produced by the NIH tetramer core facility.

To assess antigen-specific CD8+ T cell responses in C57BL/6 versus CD1d^-/-^ mice, leukocytes were isolated from mouse livers at day 14 pi and either stimulated for 5h with 10μg/ml of NrHV-NS3 or NS4 epitopes or left unstimulated (negative control) in the presence of brefeldin A (eBioscience). After 5h of incubation at 37°C cells were analyzed for intracellular IFN-γ, granzyme B and perforin as described above.

### Antibodies

The following antibodies were used for flow cytometry: purchase from Invitrogen: CD3-APC-efluor780 (145-2C11), CD4-AlexaFluor 700 (GK1.5), CD4-Pacific Orange (RM4-5), CD8-Super Bright 645 (53-6.7), NKp46-eFluor 660 or AlexaFluor 647(29A1.4), NK1.1-Super Bright 436 (PK136), GATA-3-PeCy5 (TWAJ), PD-1-SuperBright 780 (J43), Foxp3-AlexaFluor 488 (FJK-16s), CD103-AlexaFluor700 (2E7), RoRγt-PE-eFluor610 (B2D), PLZF-PeCy7 (9E12), TNF-α-PerCP-eFluor710 (MP6-XT22), IL-17-PeCy7 (eBio17B7), IL-13-Pe-eFluor610 (eBio13A), CD127-PeCy5 (A7R34), Granzyme B-eFluor450 (NGZB); purchased from Biolegend: T-bet-Brilliant Violet 605 (4B10), CD69-Brilliant Violet 711 (H1.2F3), CD44-Brilliant Violet 421 (IM7), IFN-γ-Brilliant Violet 510 (XMG1.2), TCR γδ-Pe (UC7-13D5), IL-4-Brilliant Violet 711 (11B11), IL-22-APC (Poly5164), ki67-PerCP/Cy5.5 (16A8), CXCR3-Brilliant Violet 510 (CXCR3-173), CXCR6-Brilliant Violet 421 (SA051D1), Perforin-Pe (S16009B), B220-Brilliant Violet 750 (RA3-6B2), F4/80-Brilliant Violet 785 (BM8), Ly6G/Ly6C-Alexa Fluor 700 (RB6-8C5). Appropriate isotype controls were also purchased.

The α-GalCer-CD1d-tetramer-AlexaFluor 488, the MHC class I NS4_1602-1611_ tetramer-APC and the MHC class I NS3_1043-1052_ tetramer PE were obtained from the NIH tetramer core facility.

### ALT assay

Alanine aminotransferase (ALT) levels in serum of mice were determined using an ALT activity assay Kit (Biovision) according to the manufacturers’ instructions.

### Cytometric bead array assay

IL-13, IL-4, IL-10 and IFN-γ concentrations in mouse serum were analyzed using cytometric bead array (CBA, BD Biosciences) according to the manufacturers’ instructions.

### Histology

Livers of C57BL/6 and CD1d^-/-^ mice were histologically analyzed on day 7 and 14 pi. Liver sections were fixed in 10% formalin (3-4 days) and then transferred to 70% ethanol. H&E staining of formalin-fixed mouse livers using standard procedure and pathological analysis was performed at the Einstein Histology and Comparative Pathology Core Facility.

### IL-4 and IL-13 neutralization

For the *in vivo* neutralization of IL-4 or IL-13 during acute NrHV infection mice were injected intraperitoneally with 250μg (per mouse and time point: day -1, 5, 10 and 15 post NrHV infection) of an anti-IL-4 antibody (clone 11B11, Bioxcell), an anti-IL13 antibody (clone 262A-5-1, Genentech) or a control IgG1 isotype antibody (clone HRPN, Bioxcell).

### Statistics

Statistics were calculated using GraphPad Prism (Version 9.3.1). Paired and unpaired students t test and one-way ANOVA were used to determine statistically significant differences.

## Results

### Rodent hepacivirus infection induces a heterogenous hepatic iNKT cell response

We have previously shown that acute NrHV infection in C57BL/6 mice is associated with the hepatic infiltration of lymphocyte subsets and CD4+ and CD8+ T cell dependent clearance 21-28 days post infection (pi) (28). In addition, transient antibody-mediated depletion of CD4+ T cells prior to infection leads to the establishment of chronic infection even after CD4+ T cell recovery. Mice that previously cleared primary infection are susceptible to reinfection but rapidly clear the virus within 7 days in a CD8+ T cell dependent manner (28).

To gain a better understanding into the dynamic roles of distinct lymphocyte subsets during acute and secondary infection in this novel hepacivirus mouse model we performed a high-dimensional flow cytometry analysis of lymphocyte subsets including CD4+ T cells, CD8+ cells, NK cells and NKT cells from blood, spleen and liver of C57BL/6 mice at multiple time points during infection and age-matched controls (experimental outline: **Figures S1A, B**).

**Figure 1:**
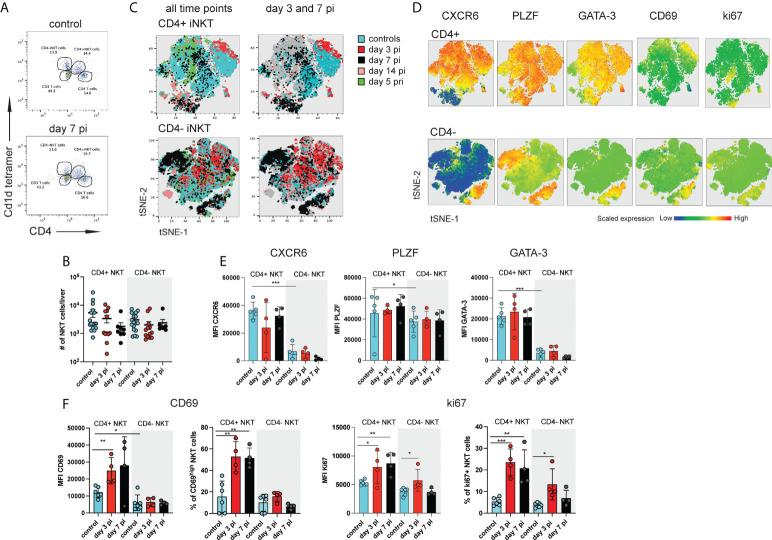
Distinct subsets of hepatic iNKT cells are activated during NrHV infection. C57BL/6 mice (n=4-8 per time point) were infected i.v. with 10^4^ genome equivalents (GE) NrHV and a flow cytometric analysis of hepatic iNKT cells was performed at multiple time points post primary infection (pi), post-secondary infection (pri) and in age-matched controls. **(A)** FACS plot showing hepatic αGalCer tet+ CD4+ and CD4- iNKT cell subsets at day 0, 7 pi. Gated on CD3+ T cells. **(B)** Total numbers of iNKT cell subsets during early NrHV infection. **(C)** tSNE representation of flow data comparing hepatic CD4+ (upper panel) and CD4- (lower panel) iNKT cells at multiple stages of NrHV infection and controls. For generation of tSNE plots data from 4 representative mice per time point was used. **(D)** Expression levels of CXCR6, PLZF, GATA-3, CD69 and ki67 in the cell populations show in **(C)**. **(E)** Mean fluorescence intensity (MFI) of CXCR6, PLZF and GATA-3 in CD4+ and CD4- iNKT cell subsets at day 3 and 7 pi and controls. **(F)** MFI and percentage of positive cells for CD69 and ki67. t test: *p< 0.5, **p<0.01, ***p<0.001.

For an initial assessment of changes in lymphocyte subsets during infection we then visualized hepatic immune cell clusters of different time points on plots generated by t-distributed stochastic neighbor embedding (t-SNE) (**Figure S1C**, acute infection). One interesting observation of our analysis was the induction of a heterogenous hepatic iNKT cell (defined as αGalCer tetramer+ CD3+ cells, see **Figure 1A**) response as indicated by the presence of iNKT cells from different time points of infection in multiple clusters on the t-SNE plot (**Figure S1C**). Total number of hepatic iNKT cells did not change during the course of infection (**Figure S1D**). In comparison, the hepatic NK cell response induced by NrHV infection was highly homogenous as indicated by the location of NK cells in only one cluster on the t-SNE plot (**Figure S1C**). Of note, while NK cells expand in the liver during acute infection [**Figure S1D** and (28)] we have previously shown that, unlike T cells, they are dispensable for acute viral clearance and do not significantly contribute to acute liver damage (28).

Given the high abundance of iNKT cells in the liver and the functional diversity of these cells we hypothesized that heterogenous iNKT cell subsets may play an important role in the hepatic immune response to hepacivirus infection.

In our initial t-SNE analysis we observed iNKT cells partially clustering with CD4+ T cells (**Figure S1C**) suggesting that CD4+ iNKT cell subsets might be activated. A detailed characterization of hepatic CD4+ and CD4- iNKT cells (**Figure 1A**) during early acute infection (day 3-14 pi; day 14 pi data not shown) and secondary infection (day 5 pri) as compared to controls showed stable number of these cells (**Figures 1B** and **S2A**) but substantial phenotypic heterogeneity as visualized on t-SNE plots (**Figures 1C** and **S2B**). Notably, CD4+ iNKT cells showed significantly higher expression of GATA-3 and PLZF as compared to CD4- iNKT cells in control mice and independent of NrHV infection (**Figures 1D, E**, **S2C**). This finding is in line with previous studies showing that GATA-3+PLZF+ type 2 (NKT2) cells are contained in the CD4+ population (15, 29). We detected similar expression patterns in splenic CD4+ and CD4- iNKT cell subsets (**Figure S3**). Hepatic CD4+ cells also presented higher expression levels of the tissue-residency markers CXCR6 and CD69 as compared to CD4- cells in the steady state (controls) (**Figures 1D–F**, **S2C**) suggesting a more pronounced tissue-residency signature in CD4+ cells. However, CD69 is also an activation marker upregulated by lymphocytes during infection. In line with this, hepatic CD4+ iNKT cells at day 3-7 pi displayed significantly higher expression of ki67 and CD69 as compared to CD4+ cells from uninfected controls indicating proliferation and activation of these cells during early acute infection (**Figures 1D, F**). CD4- NKT cells showed minor activation.

Together, our results suggest an activation of distinct iNKT cell subsets during hepacivirus infection, especially CD4+ cells during the acute phase.

### iNKT cells with a mixed type 1 and type 2 signature produce IL-4 and IL-13 during acute hepacivirus infection

**Figure 2:**
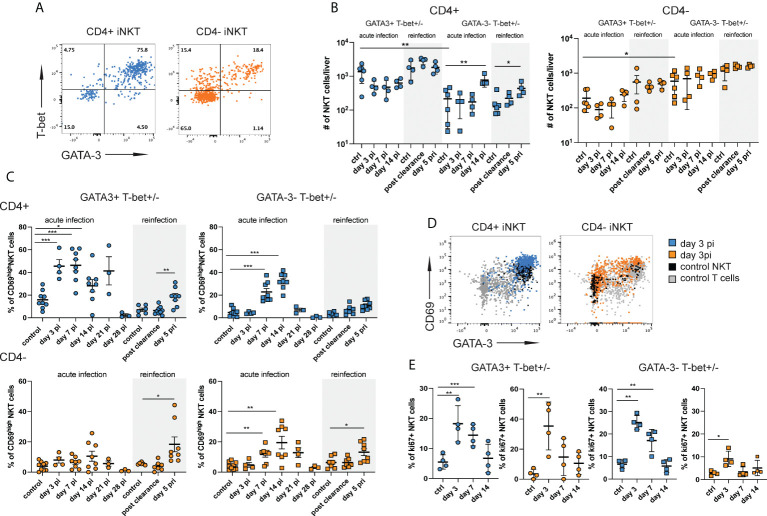
CD4+ iNKT cells with a mixed type 1 and type 2 signature are preferentially induced during acute infection. Hepatic iNKT cell subsets (n=4-8 mice per time point) were analyzed by flow cytometry at different stages of NrHV infection. **(A)** FACS plots showing T-bet and GATA-3 expression in CD4+ and CD4- subsets at day 7 pi. **(B)** Total numbers of GATA-3^+^Tbet^+^ and GATA-3^-^Tbet^+/-^ CD4+ (left graph) and CD4- (right graph) iNKT cell subsets in the liver at multiple stages of infection and controls. **(C)** CD69 expression on GATA-3^+^Tbet^+^ and GATA-3^-^Tbet^+/-^ subsets of CD4+ (upper graphs) and CD4- (lower graphs) iNKT cells. **(D)** FACS plots showing CD69 expression levels on iNKT cell subsets. **(E)** Ki67+ expression in GATA-3^+^Tbet^+^ and GATA-3^-^Tbet^+/-^ subsets of CD4+ (blue) and CD4- (orange) iNKT cells. Unpaired t test and one-way ANOVA: *p< 0.5, **p<0.01, ***p<0.001.

The mouse liver is enriched in NKT1 cells (11, 12) and we have previously shown that NrHV infection induces a strong hepatic type 1 NK cell, CD4+ and CD8+ T cell response (28). However, our initial analysis of iNKT cells (**Figure 1**) indicates that subsets of type 2 GATA-3+PLZF+ NKT cells are activated during infection. To assess the functional polarization of hepatic CD4- and CD4+ iNKT cells in more detail we analyzed T-bet and GATA-3 expression in the steady state and during acute and secondary infection. Interestingly, the majority of hepatic CD4+ iNKT cells showed high expression of both, T-bet and GATA-3, in uninfected mice suggesting a mixed NKT1/NK2 polarization of this cell subset in the steady state. In contrast, only a small fraction of CD4- iNKT cells were GATA-3+T-bet+ (**Figure 2A**). In line with this, the steady state liver contained higher numbers of GATA-3+T-bet+ than GATA-3-T-bet+/- CD4+ cells and higher numbers of GATA-3-T-bet+/- than GATA3+T-bet+ CD4- cells (**Figure 2B**). Numbers of iNKT cell subsets remained stable during infection except for a significant expansion of GATA3-Tbet-/+ CD4+ cells at day 14 pi and day 5 pri (**Figure 2B**).

GATA-3+T-bet+ CD4+ iNKT cells showed a substantial upregulation of CD69 as compared to uninfected controls starting day 3 pi indicating early activation during acute infection. GATA3-T-bet+/- CD4+ and CD4- subsets became activated starting day 7 pi (**Figures 2C, D**). All subsets also displayed high CD69 expression during secondary infection (day 5 pri, **Figure 2C**). In addition, we observed an upregulation of ki67 expression in all iNKT cell subsets suggesting proliferation during acute infection (**Figure 2E**). Relatively stable hepatic iNKT cells numbers (**Figures S1D, 1B** and **2B**) despite proliferation could suggest that most of these cells undergo activation-induced cell death at the site of infection and have a rapid turnover as described for other viral infections (30). In fact, we found significantly elevated numbers of both CD4+ and CD4- iNKT cells in the spleen (**Figure S3E**) showing that there is an expansion of iNKT cells during acute NrHV infection which is however not detectable in the liver, the site of infection. Besides a rapid turnover in the liver this might also be explained by a possible migration of iNKT cells to secondary lymphoid organs, like the spleen.

Overall, our results show the activation of a highly heterogenous hepatic iNKT cells response with the preferential early activation (day 3 pi) of CD4+ cells with a mixed type 1 and type 2 signature.

**Figure 3:**
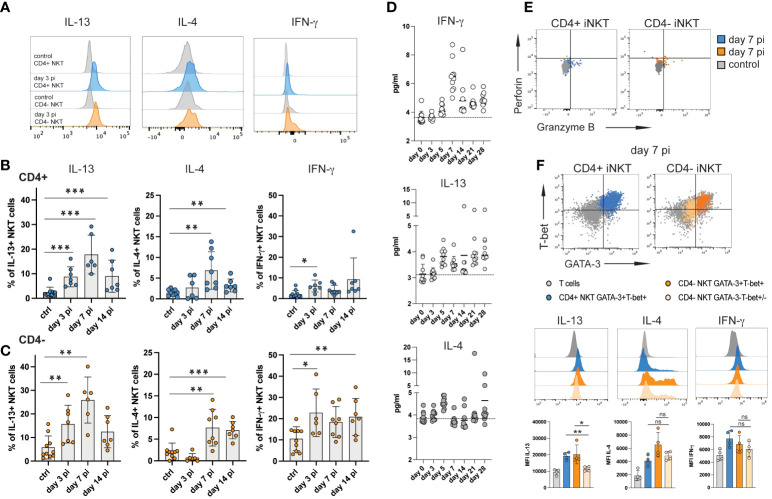
iNKT cell subsets produce type 2 cytokines during acute NrHV infection. Hepatic leukocytes were isolated from C57BL/6 mice (controls, day 3, 7, 14 pi; n=4-8 mice per time point) and immediately analyzed (no stimulation) for intracellular cytokines, granzyme B and perforin by flow cytometry. **(A)** Histograms of IL-13, IL-4 and IFN-γ expression by iNKT cell subsets at day 3 pi compared to controls. **(B, C)** Frequencies of hepatic CD4+ **(B)** and CD4- **(C)** iNKT cells producing IL-13, IL-4 or IFN-γ within the respective cell subset are shown. **(D)** Kinetics of IFN-γ, IL-13 and IL-4 serum levels during acute NrHV infection (n=10 mice) were determined by cytometric bead array. **(E)** Representative FACS plots of perforin and granzyme B expression. **(F)** Hepatic iNKT cell subsets were analyzed for the co-expression of cytokines with T-bet and GATA-3 at day 7 pi. T cells from the same time point are shown as comparison. Unpaired t test and one-way ANOVA: *p< 0.5, **p<0.01, ***p<0.001.

Next, we assessed whether the polarization and activation status of iNKT cell subsets during acute NrHV infection is associated with the expression of specific effector cytokines and molecules. Interestingly, we observed a significant production of the type 2 cytokines IL-13 and IL-4 starting day 3 pi and day 7 pi, respectively, in both, CD4+ and CD4- cell subsets as compared to controls (**Figures 3A–C**). IFN-γ was mostly expressed by CD4- NKT cells (**Figures 3A–C**) while we did not observe a production of perforin or granzyme B in any iNKT cell subset during acute infection (**Figure 3E**). Elevated levels of IL-13, IL-4 and IFN-γ could also be detected in the serum of mice during the course of acute infection (**Figure 3D**).

Splenic iNKT cells showed limited induction of cytokine production indicating that the virus-induced iNKT cell response is primarily located to the liver, the site of NrHV infection (**Figure S4**). Of note, we observed significant IL-13, but not IL-4, production also by subsets of hepatic CD4+ and CD8+ T cells (**Figure S5**) suggesting that the induction of type 2 effector functions is not limited to iNKT cells during NrHV infection.

In hepatic iNKT cells IL-13 production during infection was linked to GATA-3+T-bet+ CD4+ and CD4- subsets while IL-4 and IFN-γ was produced by GATA-3+T-bet+ as well as GATA-3-Tbet+/- cells (**Figure 3F**). NKT1 cells predominantly produce IFN-γ but can also secret IL-4 while NKT2 cells produce IL-13 and IL-4 (15). As such, the ability of GATA-3+T-bet+ cells to produce IFN-γ, IL-4 as well as IL-13 indicates that they truly represent a population with a mixed NKT1/NKT2 signature which is induced to produce predominantly type 2 cytokines during hepacivirus infection.

### iNKT cells restrain T cell effector functions and limit liver injury during acute NrHV infection

**Figure 4:**
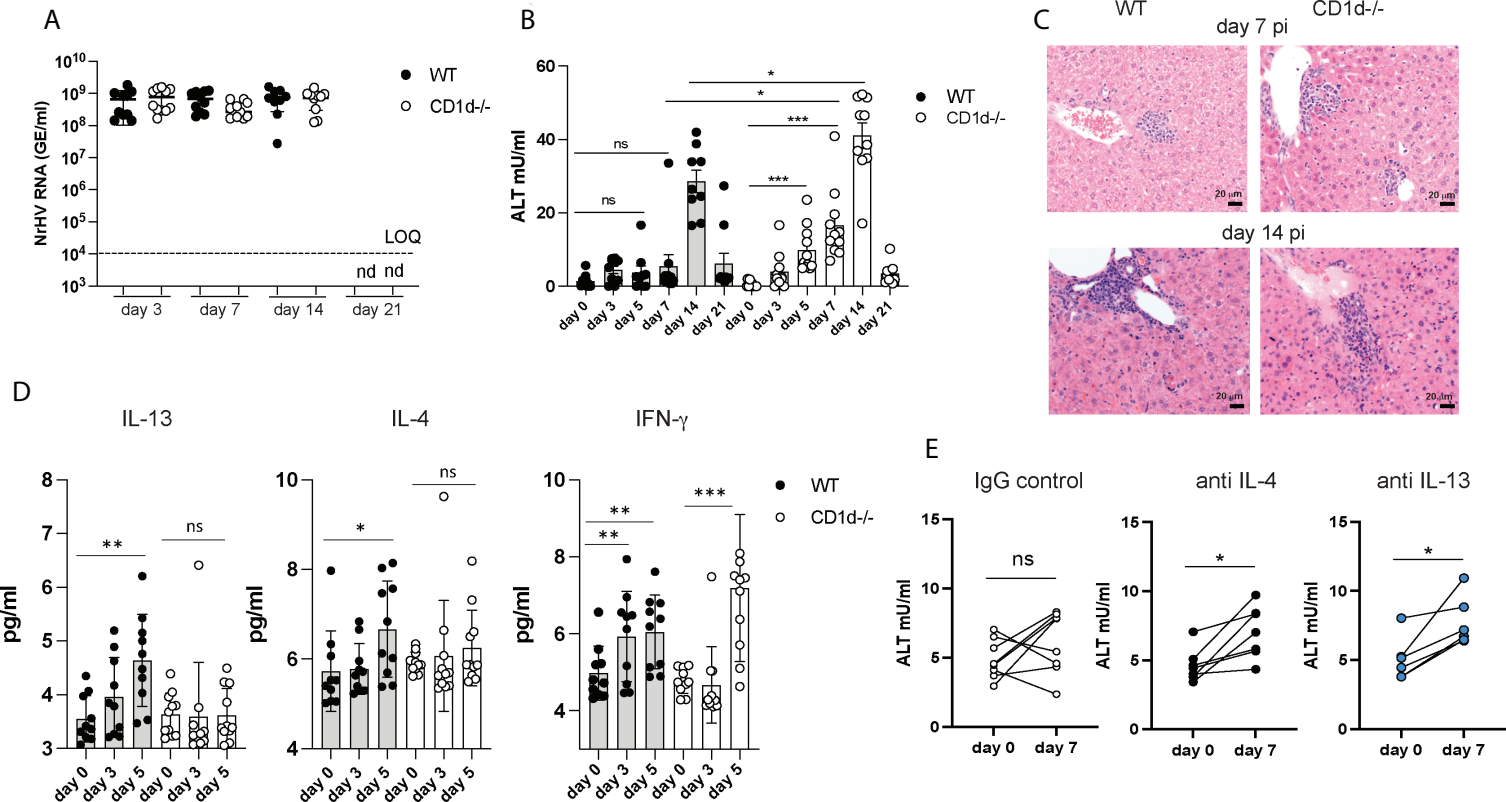
Increased liver injury in the absence of iNKT cells. C57BL/6 WT mice and CD1d^-/-^ mice (n=8-10 per group) were infected with 10^4^ GE NrHV and NrHV viremia **(A)** and ALT levels **(B)** were determined during acute infection. **(C)** Representative H&E liver histology at day 7 and 14 pi. **(D)** Serum levels of IFN-γ, IL-13 and IL-4 in WT and CD1d^-/-^ mice at days 0-5 pi were determined by cytometric bead array. **(E)** C57BL/6 mice (n=6-8 per group) were treated with neutralizing antibodies for IL-4 or IL-13 or an IgG control antibody at day -1, 5, 10 and 15 during acute NrHV infection and ALT levels were analyzed at day 0 and 7 pi. Paired and unpaired t test and one-way ANOVA: *p< 0.5, **p<0.01, ***p<0.001. ns, not significant.

To assess the direct role of iNKT cells in NrHV infection we analyzed the course of infection and extend of liver injury in NKT cell deficient CD1d^-/-^ mice as compared to C57BL/6 WT control mice. We had previously shown that acute NrHV infection is associated with immune-mediated liver injury during viral clearance in C57BL/6 mice (28). CD1d^-/-^ mice showed similar viral loads and course of acute infection (**Figure 4A**) but displayed significantly elevated alanine transaminase (ALT) levels at day 5-14 pi as compared to controls (**Figure 4B**). We also observed slightly increased leukocyte infiltration and inflammation in H&E liver histology of CD1d^-/-^ mice at day 7 and 14 pi (**Figure 4C**).

These data show that iNKT cells are dispensable for NrHV clearance which is in line with their limited production of antiviral IFN-γ, granzyme B or perforin during infection (**Figure 3**). The elevated ALT levels however suggest increased liver damage in the absence of iNKT cells and an immune-regulatory and/or tissue protective role of these cells which may potentially be mediated by their secretion of type 2 cytokines. Type 2 immunity and its effector cytokines IL-13 and IL-4 have numerous functions in the immune system which may include mediating tissue repair or regulation of type 1 immunity during a local immune response (31).

To further investigate a potential immune-regulatory role of IL-13- and IL-4- secreting iNKT cells we next determined serum cytokine levels in CD1d^-/-^ mice. Unlike in control mice (**Figures 3D**, **4D**) there was no significant increase in IL-4 or IL-13 serum levels in CD1d^-/-^ mice detectable at day 5 pi (**Figure 4D**) suggesting that iNKT cells are major producers of these cytokines during early infection. Since CD1d^-/-^ mice have functional T cells which may also produce type 2 cytokines (**Figure S5**), we analyzed iNKT cell mediated IL-4 and IL-13 secretion only at early time points before T cell activation occurs. The substantial increase in IFN-γ serum concentration at day 5 pi in both, control and CD1d^-/-^ mice, (**Figure 4D**) is likely mediated by NK cells which infiltrate the liver and produce IFN-γ during early NrHV infection (28).

Groups of C57BL/6 mice treated throughout infection with neutralizing antibodies for either IL-4 or IL-13, but not mice treated with an IgG control, showed elevated ALT levels at day 7pi (**Figure 4E**) suggesting a direct role of these cytokines in limiting liver injury during early acute infection. However, the effect was less significant as observed in CD1d^-/-^ mice. This could be due to redundant functions of IL-4 and IL-13, incomplete neutralization of these cytokines or additional IL-4/IL-13 independent iNKT cell functions.

**Figure 5:**
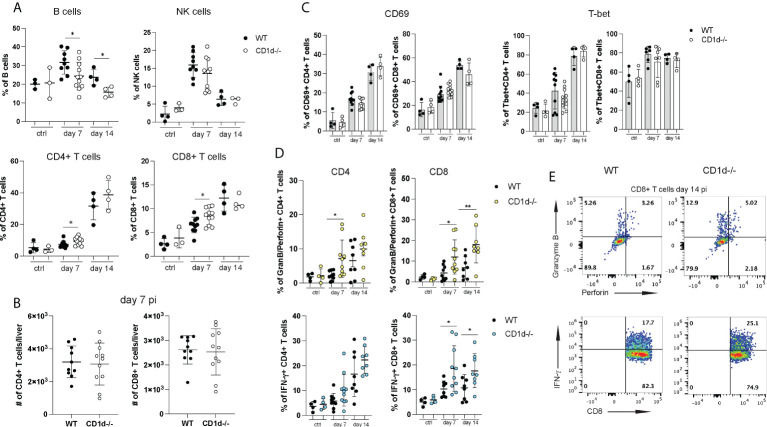
Hepatic lymphocyte subsets in CD1d^-/-^ mice during acute infection. A flow cytometric analysis of hepatic lymphocytes in WT and CD1d^-/-^ mice (n=4-8 per time point) was performed at day 7 and 14 pi and in uninfected controls. **(A)** Frequencies of B cells, NK cells, CD4+ and CD8+ T cells. **(B)** Total numbers of hepatic CD4+ and CD8+ T cells at day 7pi. **(C)** CD69 and T-bet expression of CD4+ and CD8+ T cells. **(D)** Granzyme B and perforin (upper graphs) and IFN-γ (lower graphs) expression of T cell subsets after unspecific stimulation with PMA/Ionomycin. **(E)** Representative FACS plots of granzyme B/perforin and IFN-γ expression in CD8+ T cells at day 14 pi. Unpaired t test: *p< 0.5, **p<0.01.

Liver injury during acute NrHV clearance is primarily mediated by CD8+ T cells but not by NK cells (28). To assess if iNKT cells play a role in the regulation of the antiviral T cell response we analyzed hepatic immune cell subsets in CD1d^-/-^ mice and WT controls at day 7 and 14 pi. CD1d^-/-^ mice showed lower hepatic frequencies of B cells but higher T cell frequencies (**Figure 5A**) as compared to controls while the frequencies and total cell numbers of NK cells, regulatory T cell and myeloid cell (**Figure 5A** and data not shown) were comparable. Total CD4+ and CD8+ T cell numbers infiltrating the liver at day 7pi (**Figure 5B**) and day 14 pi (data not shown) did also not differ in the absence of iNKT cells. In our previous study we showed that NrHV infection leads to a strong activation of a hepatic type 1 CD4+ and CD8+ T cell response starting around day 7pi (28). CD69, T-bet (**Figure 5C**), CD44 and Ki67 (data not shown) upregulation in T cells was similar in CD1d^-/-^ mice and controls. However, CD4+ and CD8+ T cells of CD1d^-/-^ mice isolated at day 7-14 pi and unspecifically stimulated with PMA/Ionomycin showed significantly increased production of IFN-γ and granzyme B and perforin (**Figures 5D, E**) as compared to controls. Together, this analysis shows that a lack of iNKT cells does not considerably affect overall hepatic immune cell infiltration or activation during NrHV infection. However, these cells may modulate T cell effector functions.

**Figure 6:**
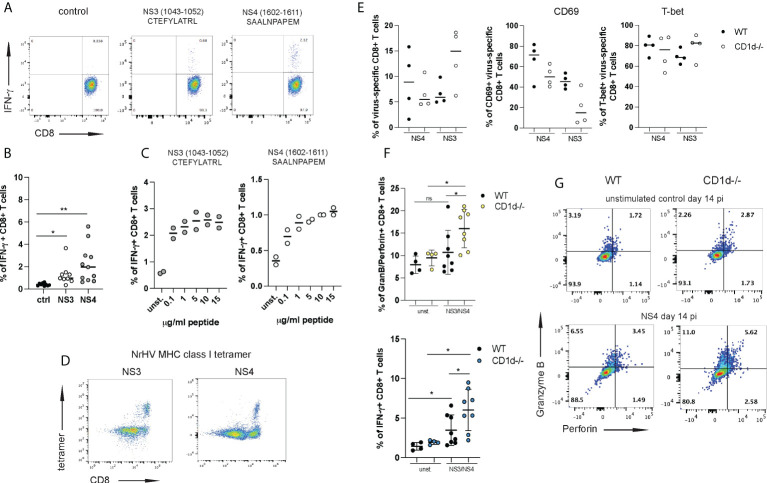
iNKT cells restrain antiviral effector functions of virus-specific CD8+ T cells. Two immune-dominant MHC class I restricted NrHV epitopes were identified using an overlapping NrHV peptide library. **(A)** Representative FACS plots showing intracellular IFN-γ production by hepatic CD8+ T cell isolated at day 21 pi and stimulated with 10ug/ml of the NrHV NS3 or NS4 epitope for 5h. Gated on CD8+ T cells. **(B)** Summarized data of **(A)**. **(C)** IFN-γ production by CD8+ T cells in response to stimulation with increasing concentrations of the NS3 or NS4 peptide. **(D)** FACS plots showing hepatic NS3- or NS4- specific CD8+ T cells at day 21 pi using NS3- or NS4- MHC class I tetramer staining. Gated on CD3+ T cells. **(E)** Frequencies of NS3- and NS4-specific cells within the hepatic CD8+ T cell population in WT and CD1d^-/-^ mice (n=4 per group) at day 14 pi (left graph) and CD69 and T-bet expression on NS3- and NS4-specific CD8+ T cells (right graphs) at day 14 pi. **(F-G)** CD8+ T cells were isolated at day 14 pi from WT and CD1d^-/-^ mice (n=4 per group), stimulated with the NS3 or NS4 peptide or left unstimulated and analyzed for antigen-specific granzyme B/perforin and IFN-γ expression. Summarized data (NS3 and NS4 combined) is shown in **(F)** and representative FACS plots in **(G)**. Unpaired t test: *p< 0.5, **p<0.01. ns, not significant.

To further investigate this possibility, we set out to analyze the effector functions of NrHV-specific CD8+ T cells. We had previously reported significant IFN-γ production by CD8+ T cells after stimulation with peptide pools of the NrHV nonstructural proteins NS3 and NS4 (28). To identify minimal MHC class I restricted (C57BL/6 background) epitopes in the viral genome we then used an overlapping peptide library covering the entire NrHV clone B genome in combination with IFN-γ Elispot, epitope prediction (Immune Epitope Data Base, IEDB) analysis (data not shown) and intracellular cytokine staining (**Figure 6A**). With this approach, we identified two CD8+ T cell epitopes in NS3 and NS4: H2Kb restricted NS3_1043-1052_ CTEFYLATRL and H2Db restricted NS4_1602-1611_ SAALNPAPEM (**Figure 6A**). Hepatic CD8+ T cells isolated at day 21 pi and stimulated *in vitro* for 5h with different concentrations of the NS3 or the NS4 epitope showed significant antigen-specific IFN-γ production (**Figures 6B, C**). Functional tetramers to detect NS3- and NS4-specific CD8+ T cells were generated with these epitopes (**Figure 6D**). Using these new tools, we then compared virus-specific CD8+ T cell responses in CD1d^-/-^ and WT controls at day 14 pi. Frequencies of NS3- or NS4-specific cells within the hepatic CD8+ T cell population were highly variable in individual mice with no statistically significant difference between CD1d^-/-^ and control mice (**Figure 6E**). Interestingly, we observed a downregulation of CD69 on virus-specific CD8+ T cells from CD1d^-/-^ mice while T-bet expression was comparable in both groups of mice (**Figure 6E**). In line with the analysis of global T cells (**Figure 5**) the most pronounced differences between virus-specific CD8+ T cells from CD1d^-/-^ and control mice were associated with their effector functions. In fact, we observed significantly elevated NS3- and NS4-specific granzyme B/perforin and IFN-γ production in the absence of iNKT cells (**Figures 6F, G**). These results further confirm a role of hepatic iNKT cells in restraining inflammatory and cytotoxic effector functions of antiviral T cells. Interestingly, we observed elevated serum concentrations of the immune-regulatory cytokine IL-10 during NrHV infection in both, WT and CD1d^-/-^ mice (**Figure S6**). However, IL-10 levels were significantly reduced at day 14 pi in mice lacking NKT cells as compared to controls. This finding may indicate that iNKT cells exert regulatory functions through IL-10, either by directly producing IL-10 or by acting on IL-10 producing cells, such as regulatory T cells. Regulatory and tissue protective roles for iNKT cells through type 2 cytokine or IL-10 production and expansion of regulatory T and B cells have been described in recent studies investigating iNKT cell subsets in other model systems (25, 32–34).

In conclusion, our study suggests that iNKT cells skewed towards type 2 immunity, either directly or indirectly, limit acute liver injury during hepaciviurs infection by mechanisms that include regulating the antiviral effector functions of virus-specific T cells.

## Discussion

In this study we used a novel rodent hepacivirus mouse model to characterize hepatic iNKT cell subsets during different stages of an HCV-related virus infection *in vivo.* We report the activation of a highly diverse iNKT cell response that was associated with an activation of iNKT cells exhibiting a mixed NKT1/NKT2 signature and production of the type 2 cytokines IL-4 and IL-13. The phenotypical and functional properties of hepatic iNKT cells during HCV infection, and viral hepatitis in general, are not well defined especially during acute infection (12, 19). A recent study reported an activation of peripheral iNKT cells that correlated with liver damage during acute HCV infection in human patients suggesting that these cells play a role in acute viral hepatitis. However hepatic cells were not analyzed in this study (21).

Thus, our study provides significant new insight into the potential function of these cells in the liver during a hepatotropic virus infection.

It has been described that the majority of iNKT cells in the mouse liver are NKT1 cells (11, 13, 15) and that these cells can mediate anti-tumor immunity in liver cancer (35) and antiviral immunity in a mouse model of HBV infection (18, 36). In line with the reported NKT1 polarization of hepatic iNKT cells we observed high expression of T-bet in CD4+ and CD4- iNKT cells. However, we also showed that a significant number of these cells, in particular CD4+ cells, co-express GATA-3 with T-bet in the steady state and during infection. GATA-3+T-bet+ cells produced IFN-γ, IL-4 and IL-13 which indicates a mixed NKT1/NKT2 signature. In line with our findings, a recent study reported that hepatic iNKT cells largely represent a cell subset co-expressing certain transcription factors including T-bet and GATA-3 and exhibiting a high phenotypic and functional plasticity (25). In addition, hepatic iNKT cells with a type 2 polarization have been described by other studies. For example, a role for IL-4 secreting iNKT cells in sterile liver injury (22) or the induction of NKT1/NKT2 cells in mouse and human NASH (37) have been reported. Divergent findings on the phenotypical and functional properties of hepatic iNKT cells may be explained by the high functional diversity and plasticity of these cells (17, 38). In fact, iNKT cells can rapidly respond to cytokines released in their specific tissue microenvironment even without TCR engagement (15, 16). It is thus conceivable that the hepatic immunological microenvironment during a specific immune response differentially shapes the polarization and function of iNKT cells.

Our study describes the activation of NKT1/NKT2 cells producing IL-4 and IL-13 during an acute hepatotropic virus infection. Type 2 immunity is not typically associated with antiviral immunity and in line with this iNKT cells were dispensable for NrHV clearance. However, type 2 immune responses can be induced to mediate immune-regulation or facilitate tissue protection and repair during inflammatory type 1 immunity (31). Indeed, our finding of increased acute liver injury in the absence of iNKT cells supports an immune-regulatory and/or tissue protective role of these cells and potentially their type 2 effector cytokines during hepacivirus infection. In the NrHV mouse model antiviral CD8+ T cells are main mediators of acute liver injury (28). In this study we identified two novel CD8+ T cell NrHV epitopes which allowed us to assess the impact of iNKT cells on the functional properties of virus-specific CD8+ T cells. Our finding that the absence of iNKT cells leads to significantly elevated antiviral effector functions in hepatic CD8+ T cells suggests a role of iNKT cells in limiting liver injury by regulating the antiviral T cell response. While our study was focused on the T cells it is also possible that iNKT cells modulate other immune cell subsets with antiviral and cytotoxic potential such as NK cells.

Of note, CD1d^-/-^ mice are also deficient in non-invariant type II NKT cells (19). While these cells are not the focus of the current study and are not the major hepatic NKT cell subset it is possible that they may contribute to the observed phenotype in CD1d^-/-^ mice. Clearly, additional studies are needed to determine the direct or indirect mechanisms of iNKT cell mediated T cell regulation and the role of IL-4 and IL-13. It will also be interesting to assess if IL-4 or IL-13 secreted by iNKT cells directly contribute to tissue repair as has been shown in a model of sterile liver injury (22). Further we plan to investigate a possible link between type 2 iNKT cells, IL-10 and regulatory T cells during NrHV infection since we observed lower IL-10 levels in CD1d^-/-^ mice and have previously reported regulatory T cell expansion during NrHV infection (28). Related to that possibility, it was recently reported that hepatic iNKT cells can develop into IL-10 producing cells that activate IL-10+ regulatory B cells to suppress autoreactive T cells (25).

Type 2 cytokine production during acute NrHV infection was not limited to iNKT cells but was also observed in CD4+ and CD8+ T cells subsets. Interestingly, a recent study showed that IL-13 secreting innate-like CD8+ T cells promote tissue repair during skin injury (39). While the phenotypical and functional properties of type 2 T cell subsets during hepacivirus infection still need to be analyzed in detail it is possible that IL-4+/IL-13+ iNKT cells, CD4+ and CD8+ T cells have certain functional redundancy. This may explain the limited effects we observed in NKT cell deficient mice. Thus, the iNKT cells we characterized in this study may represent one cell subset of a broader type 2 immune response induced to limit acute liver pathology during the antiviral type 1 immune response. Future studies using conditional knock-out mouse strains for specific cell subsets and/or cytokines should reveal individual or redundant functions of type 2 iNKT cells, T cell subsets and the effector cytokines IL-13 and IL-4.

During a chronic immune response type 2 immunity can be a significant driver of fibrosis development (40). As such it would be interesting to also assess a potential role of type 2 iNKT cells or T cell subsets during the chronic stages of NrHV infection. Of note, a recent study identified a subset of IL-4 producing CD4+ T cells in the human liver that correlated with necroinflammatory scores in chronic HBV patients (41).

Another question that arises from our study is the mode of iNKT cell and type 2 immunity induction during acute NrHV infection. Infected hepatocytes or other hepatic cells can secret alarmins such as IL-33 or IL-25 which may directly polarize iNKT cells to secrete type 2 cytokines (31, 40). Another possibility is altered self-lipid presentation on CD1d by virus-infected hepatocytes and activation of iNKT cells through TCR signaling which has been shown to induce NKT cells in a mouse model of HBV infection (18). Single-cell RNA sequencing analysis of hepatic cell subsets, including infected hepatocytes, during early acute infection may reveal transcriptomic signatures that could help to determine the exact mode of iNKT cell activation and type 2 polarization during hepacivirus infection *in vivo*.

In conclusion, we used a novel mouse model of an HCV-related rodent hepacivirus that allows to study the role of specific hepatic lymphocyte subsets during acute infection. This type of study was previously impeded due to a lack of a suitable animal model and extremely limited availability of human liver tissue during acute HCV infection. Our study revealed a novel role for iNKT cells with a mixed NKT1/NKT2 phenotype and type 2 cytokine secretion in limiting liver injury during acute infection by regulating the effector functions of antiviral T cells. In addition, we identified two immune-dominant CD8+ T cell viral epitopes which will be useful in characterizing virus-specific hepatic CD8+ T cell responses in any future study using this model.

The results from our study contribute to a better understanding of hepatic immune regulation during a hepatotropic virus infection *in vivo* and may have implications for HCV vaccine development or the advance of immune-therapeutic strategies for liver disease.

## Data availability statement

The original contributions presented in the study are included in the article/**Supplementary Material**. Further inquiries can be directed to the corresponding author. 

## Ethics statement

The animal study was reviewed and approved by IACUC, Albert Einstein College of Medicine.

## Author contributions

SR and EB conceived the project and designed experiments. SR and JL-S performed experiments. SR, JL and EB analyzed data. EB wrote the manuscript. All authors read and edited the manuscript. All authors contributed to the article and approved the submitted version.

## Funding

For this study EB received funding through a Pinnacle Research Award in Liver Disease from the American Association for the Study of Liver Diseases (AASLD) Foundation, through start-up funds from Albert Einstein College of Medicine, through NIH (National Institutes of Health) grant R01AI170725 and a pilot grant from NIH Liver Center grant P30DK041296-31. Additional funding was received through NIH grant R01AI131688 (awarded to Charles M. Rice). JL-S. received funding through NIH T32 training grant 5T32GM007491. NIH Cancer Center support grant P30CA013330 partially supports all work conducted through the shared facilities at Einstein, including the Flow Cytometry core and the Histology and Comparative Pathology core.

## Acknowledgments

We would like to thank Charles M. Rice for support, Troels K.H. Scheel and Raphael Wolfisberg for help in generating the NrHV virus stock and the members of the Einstein Flow Cytometry Core Facility for technical advice. The tetramers used in this study were provided by the NIH tetramer core facility.

## Conflict of interest

Author JL was employed by FlowJo.

The remaining authors declare that the research was conducted in the absence of any commercial or financial relationships that could be construed as a potential conflict of interest.

## Publisher’s note

All claims expressed in this article are solely those of the authors and do not necessarily represent those of their affiliated organizations, or those of the publisher, the editors and the reviewers. Any product that may be evaluated in this article, or claim that may be made by its manufacturer, is not guaranteed or endorsed by the publisher.
