# Supplementary figures and images for: Hepatic iNKT cells produce type 2 cytokines and restrain antiviral T cells during acute hepacivirus infection
Source: Front Immunol. 2022 Sep 9;13:953151. doi: 10.3389/fimmu.2022.953151 (PMC9501689; doi:10.3389/fimmu.2022.953151)

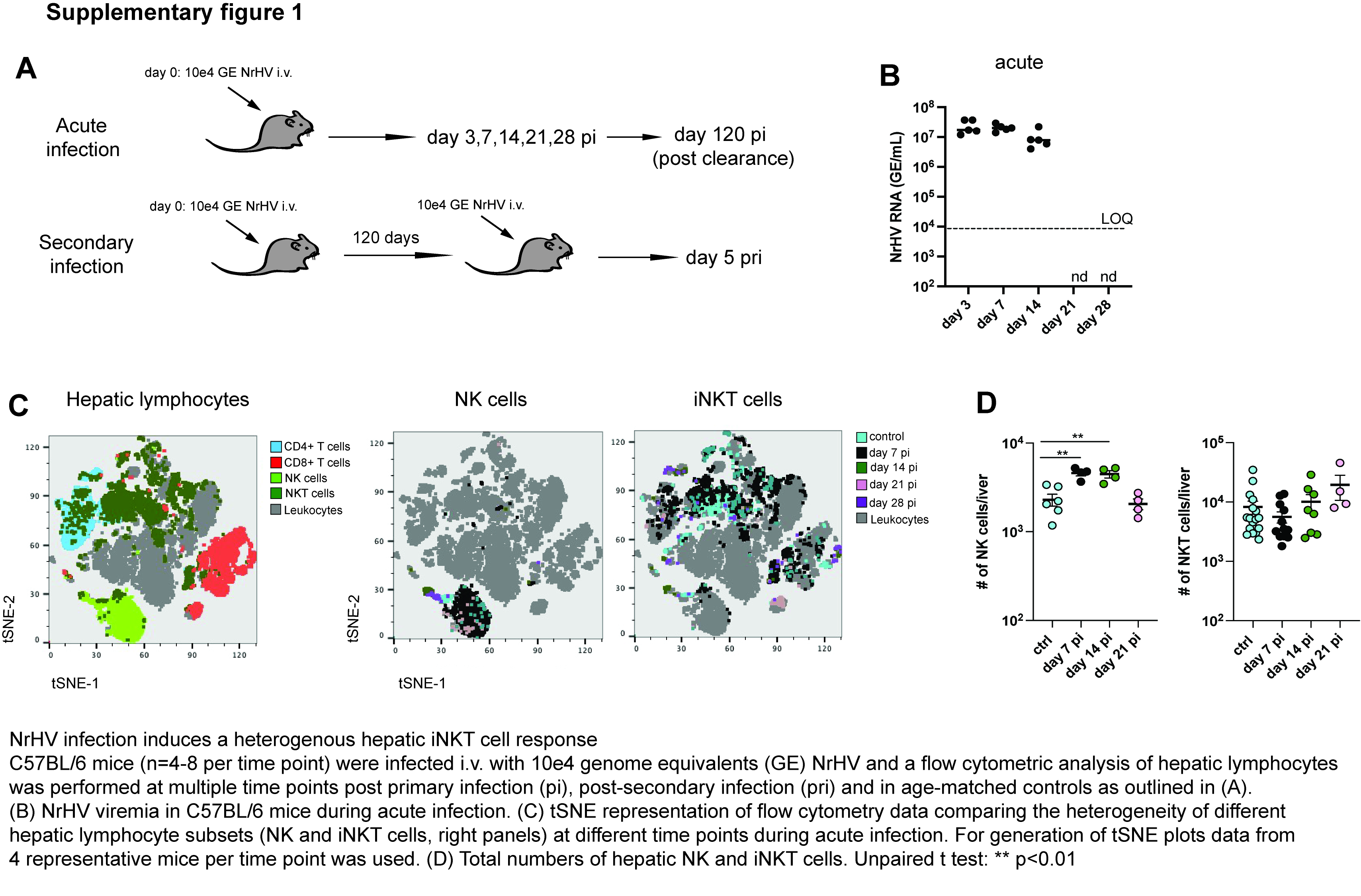

Supplement: Supplementary file 1 [file Image_1.tif]

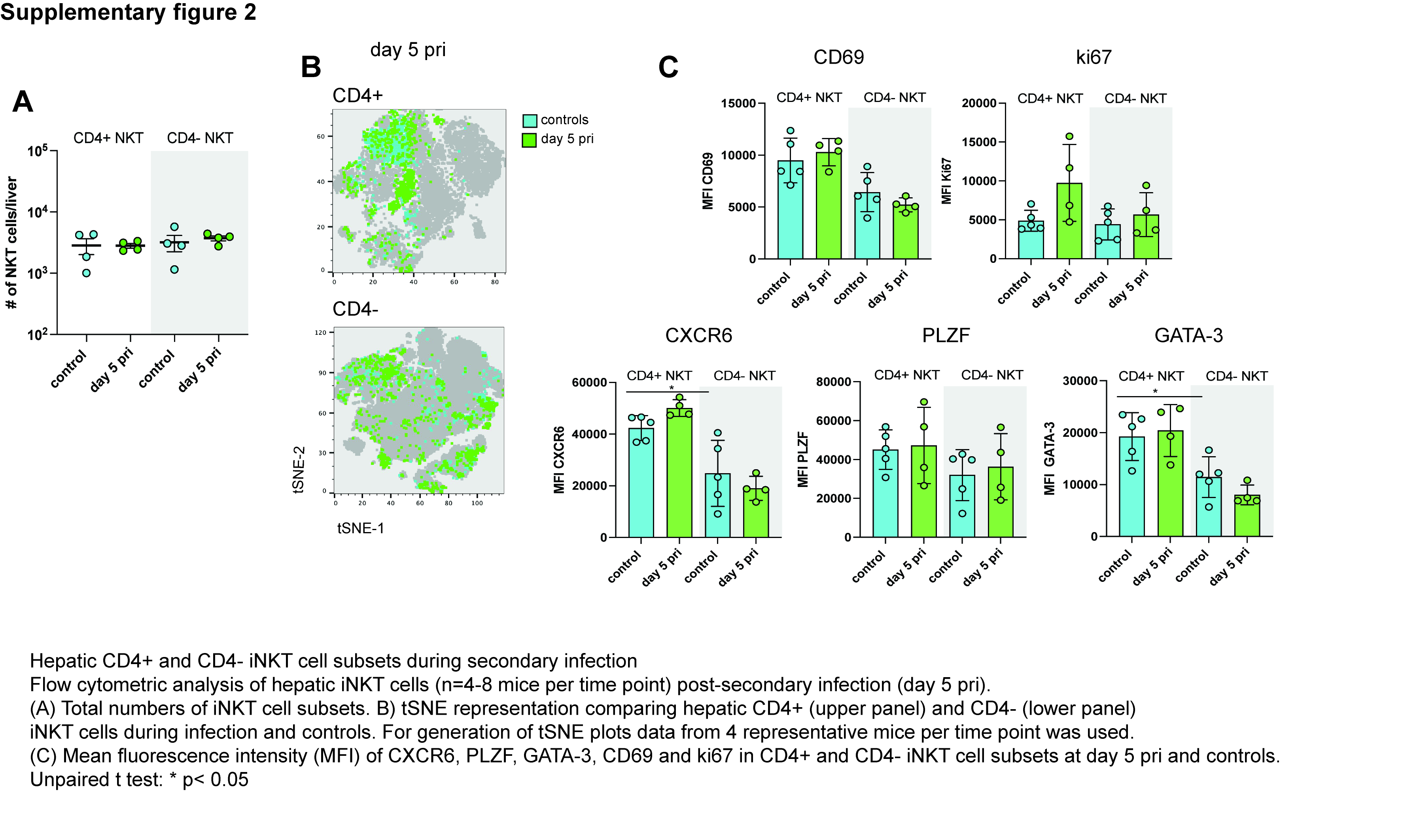

Supplement: Supplementary file 2 [file Image_2.tif]

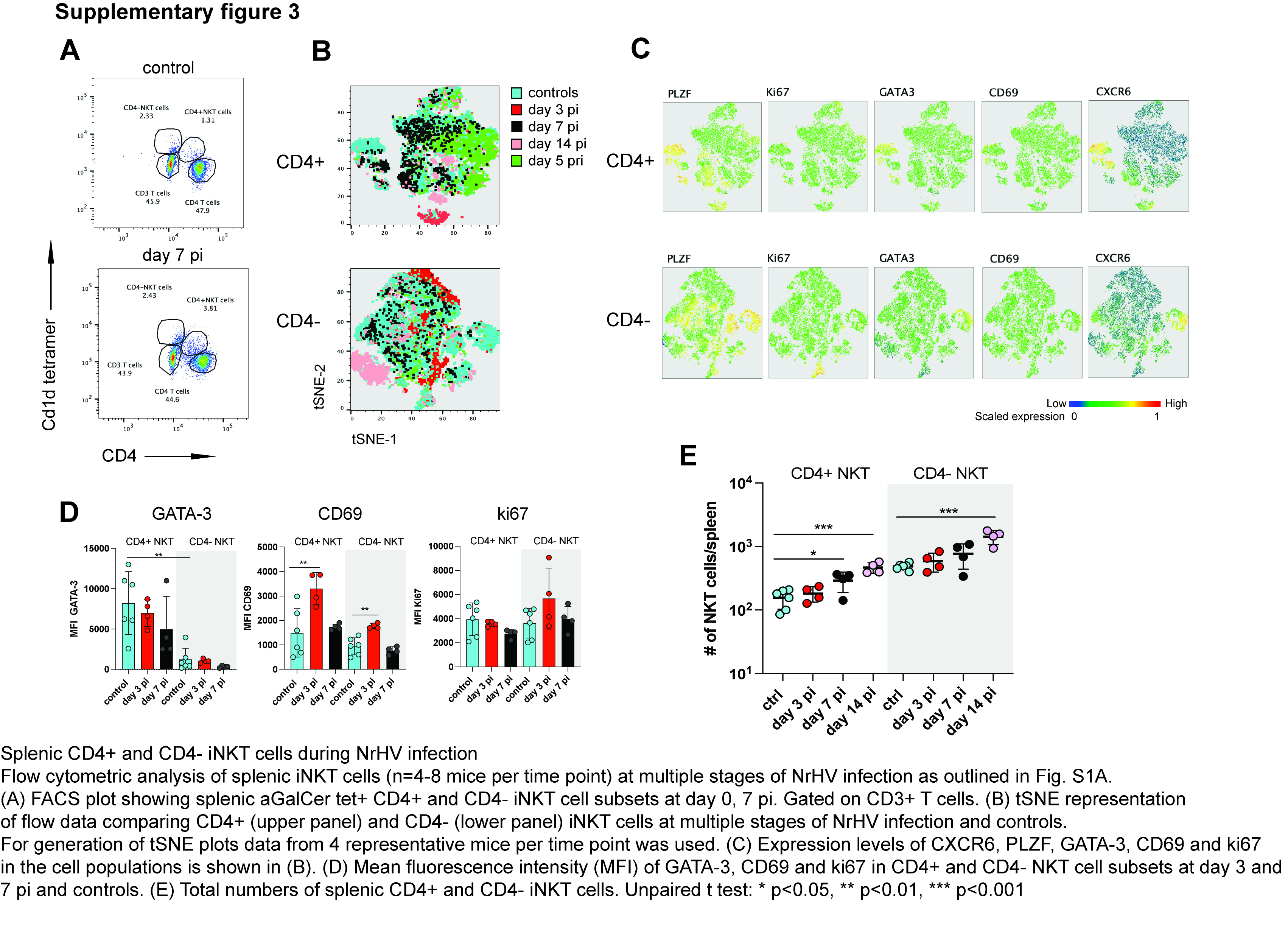

Supplement: Supplementary file 3 [file Image_3.tif]

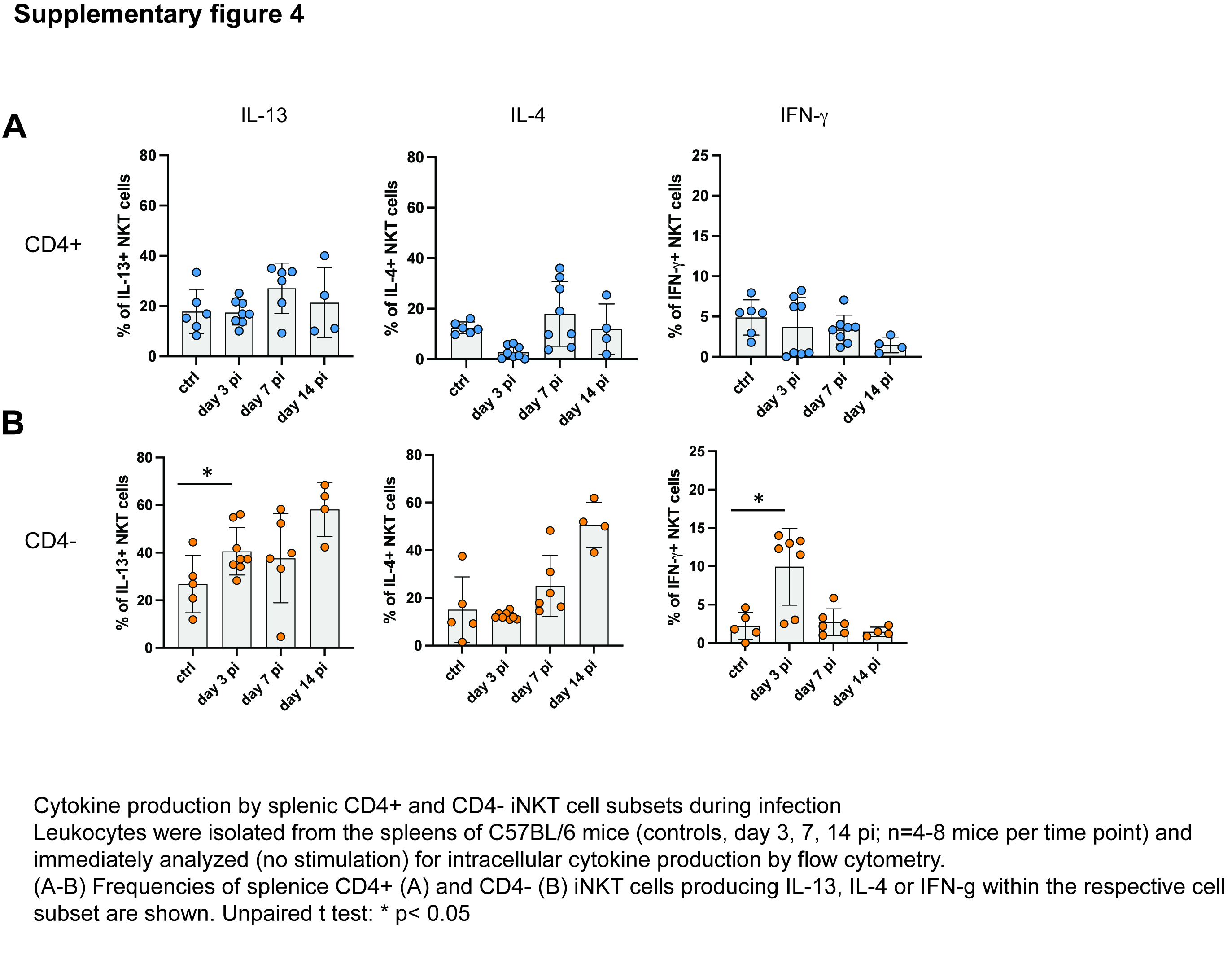

Supplement: Supplementary file 4 [file Image_4.tif]

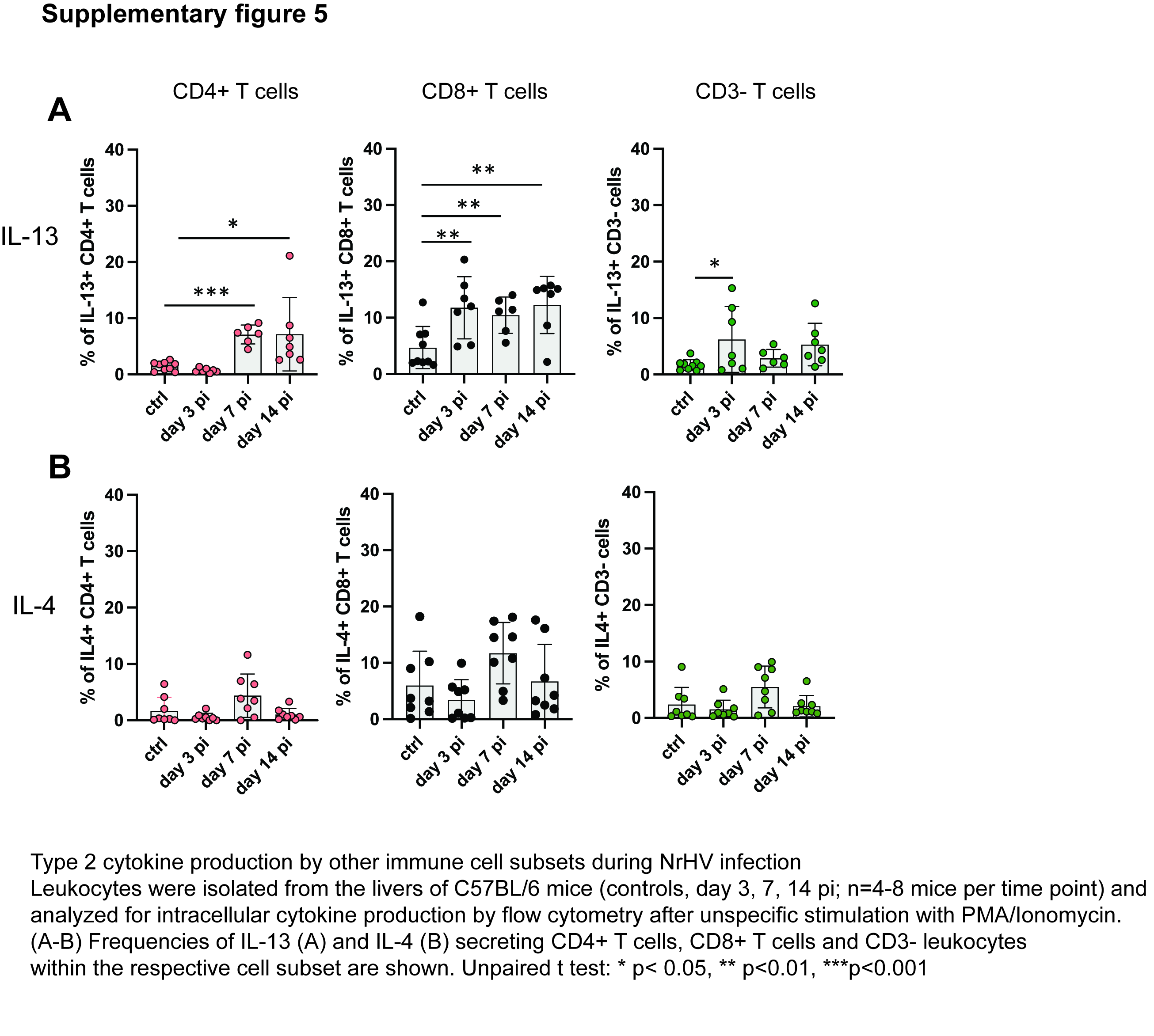

Supplement: Supplementary file 5 [file Image_5.tif]

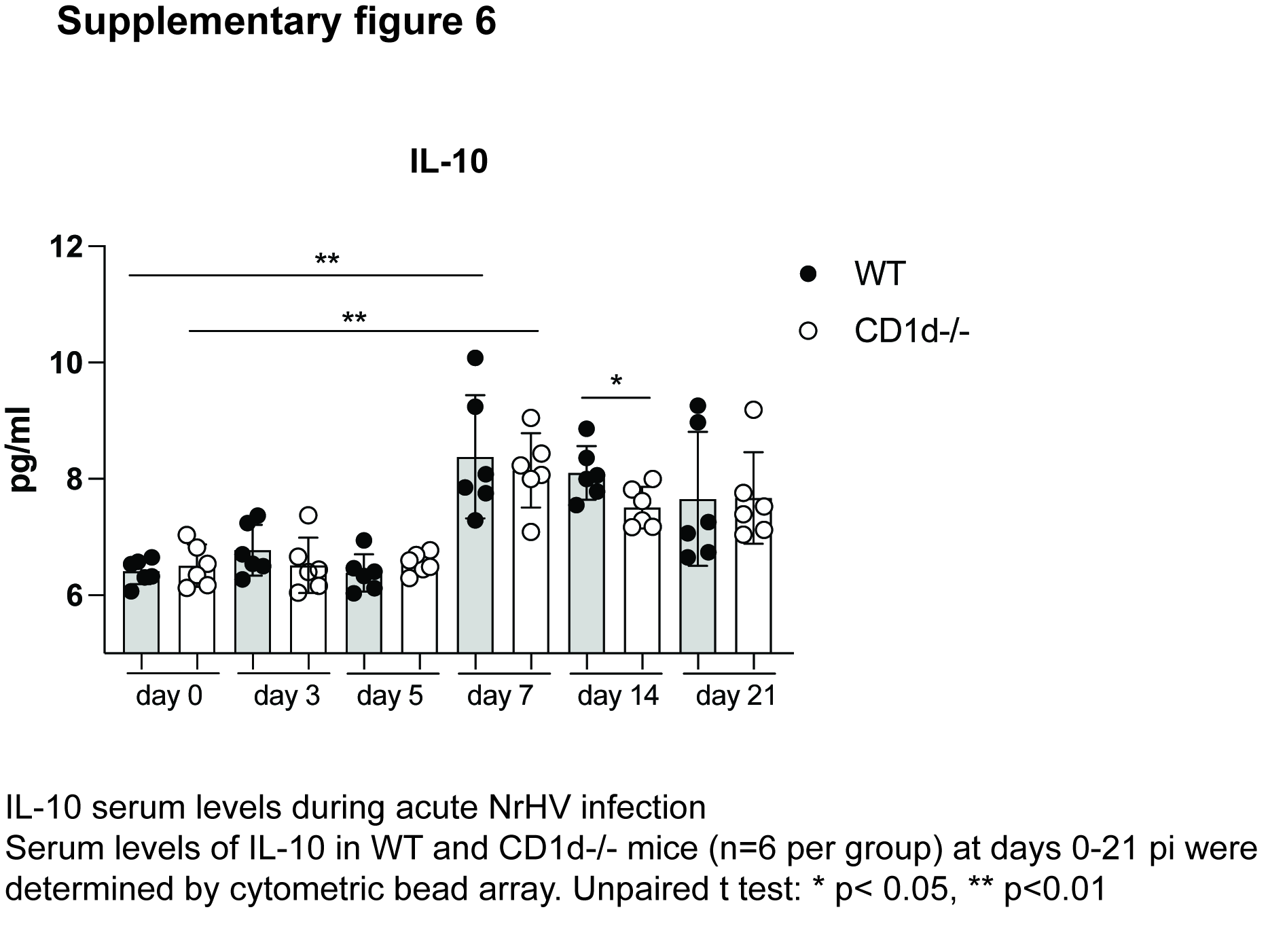

Supplement: Supplementary file 6 [file Image_6.tif]
